# Stroke thrombolysis given by emergency physicians cuts in-hospital delays significantly immediately after implementing a new treatment protocol

**DOI:** 10.1186/s13049-016-0237-0

**Published:** 2016-04-11

**Authors:** Iiro Heikkilä, Hanna Kuusisto, Alexandr Stolberg, Ari Palomäki

**Affiliations:** Department of Emergency Medicine, Kanta-Häme Central Hospital, Ahvenistontie 20, FI-13530 Hämeenlinna, Finland; Department of Neurology, Kanta-Häme Central Hospital, Ahvenistontie 20, FI-13530 Hämeenlinna, Finland; School of Medicine, University of Tampere, FI-33014 Tampere, Finland

**Keywords:** *Acute ischaemic stroke*, *Emergency medicine*, *Thrombolysis*, *reorganization*, *Emergency department*

## Abstract

**Background:**

Tissue plasminogen activator (tPA) treatment for acute ischaemic stroke (AIS) should be given as soon as possible, preferably within 60 min after arrival at hospital. There is great variation in door-to-needle times (DNTs) internationally, nationally and even within the same hospital. Various strategies for improving treatment delays have been presented. The role of emergency physicians (EPs) in treating AIS has been under discussion in recent years. Emergency Medicine (EM) officially became a specialty in Finland in 2013. Practical education of EPs in Kanta-Häme Central Hospital began in October 2012, together with reorganization of the in-hospital treatment path for AIS patients. The main change was shifting the on-call duty regarding stroke patients from internists or neurologists to EPs after the third quarter of 2013.

**Methods:**

This was a retrospective study. The data, concerning the characteristics of tPA-treated patients, DNTs and onset-to-treatment times (OTTs) was collected from electronic and paper records. The period studied was 1 year before and 1 year during reorganization, i.e. 2012 and 2013.

**Results:**

During the study period a total of 64 tPA treatments were given, 31 before and 33 during reorganization. The median DNT was 54 min in 2012, while it was 28 min in 2013 (*p* < 0.001). The median OTTs were 139 and 101 min before and during the start of reorganization, respectively (*p* < 0.001).

**Conclusions:**

Both total and in-hospital delays in the treatment of ischaemic stroke were shortened significantly during reorganization. Emergency physicians are able to treat AIS patients within international time guidelines. Success was based on scrutinized reorganization and good cooperation between neurologists, EPs and radiologists.

## Background

According to international guidelines regarding acute ischaemic stroke (AIS), recombinant tissue plasminogen activator (tPA) should be given within 4.5 h after the onset of symptoms and within 60 min after patient arrival at an Emergency Department (ED) in order to obtain maximum benefit from the treatment [1]. As commonly cited, “time is brain”, meaning the sooner that tPA is given, the better the benefit [2, 3]. Recently the Stroke Unit of Helsinki University Hospital (HUCH) launched a systematic protocol to reduce door-to-needle times (DNTs) to 20 min [4]. HUCH is offering a telemedicine service (also known as “telestroke”) to secondary-care hospitals that cannot arrange full-time on-site stroke evaluation [5]. It has been estimated, that the Finnish rate of thrombolysis in year 2007 was 6 % of all ischaemic strokes [6].

Kanta-Häme Central Hospital (KHCH) is a secondary-care hospital located in Southern Finland. The catchment population is 175 000. Before 2013, the evaluation protocol for a stroke patient was different depending on the day of the week and the time of day. During office hours and at weekends neurologists or resident neurologists were responsible for the evaluation and treatment of AIS patients. From Monday to Thursday 4–10 p.m., resident internists on call took care of AIS patients with the help of telestroke. At night there was no telestroke service available and thrombolysis candidates were transported about 100 km to the nearest university hospital by the emergency medical services (EMS). This system varied a little over time during 2009–2012 and was quite complex and hard to assimilate. For example there were many different phone numbers to responsible physicians depending on the weekday. This caused discomfort especially among nurses and EMS.

Emergency Medicine (EM) officially became a specialty in Finland at the beginning of 2013. However, practical education of Emergency Physicians (EPs) started earlier in our hospital, in October 2012 [7]. It was preceded by systematic education and practical training of nurses working in the ED [8]. Since there was a relative lack of neurologists on call in KHCH, we decided to create a new protocol for treating stroke patients, relying on EPs. During the year 2013 the number of resident EPs involving the process was totally nine. Their clinical experience in the ED varied between 2.5 to 6 years. The other staff in the protocol includes ED nurses, laboratorians, radiographers, radiologist and senior neurologist on-call if needed.

In this article we describe a new protocol for AIS patients and thrombolysis given by EPs in the ED-the so-called Hämeenlinna model. We also present preliminary results concerning DNTs and onset-to-treatment times (OTTs).

## Methods

This retrospective study consists of two parts. First, we present our reorganized treatment path for AIS patients in the ED, which is based on scrutinized teamwork led mainly by an EP. In the second part we deal with analyses of in-hospital delays and total symptom onset-to-treatment time of AIS patients. Patients were treated according to international and national guidelines [1]. The study was approved by the local ethics committee.

### Reorganization

When EM became its own specialty in Finland at the beginning of 2013, the evaluation and treatment protocol of AIS was reorganized in such a way that the primary responsibility was on EPs, which made it possible to give up the telestroke service. The model includes strategies presented in Table 1 [4, 9, 10].

**Table 1:** Actions involved in the new protocol

Facilities and education		
*Measure*	*Description*	*Done or not*
Build a new specialty	EM became its own specialty in 2013	Done
Education, including regular feedback on DNT [10]	Educate EPs in treatment of AIS	Done
Face the facts with ED staff	Have a collective target-improving practice.	Done
Have good cooperation between specialties	Good cooperation between EM, neurology, radiology	Done
Reorganize and involve the EMS [4]	EMS and ED management on same wavelength. Education of EMS personnel	Done
Pre-hospital		
Pre-notification from EMS [4, 9]	Alarm from EMS to ED triage, target 15 min before arrival	Done
Single call activation system [9]	Triage alerts physician and nurses at the same time	Done
Patient history before arrival [4]	Physician explores patient medical history from patient records if available	Done
Alarm and pre-order of tests [4]	Laboratory and CT referrals done at pre-notification	Done
In-hospital		
Face the patient in the ED lobby; whole stroke team present	Patient examined upon arrival at the ED lobby on the EMS bed	Done
POC INR [4]	INR measured while physician examines the NIHSS	Done
CT relocated to ER [4]	CT located next to lobby	Done
CT priority / CT with no delay [9]	Free the CT table from unnecessary studies	Done
Radiologist available 24/7	Oral or written report on CT available in less than 5 min	Done
tPA stored in ED [10]	tPA stored in primary care room	Done
Premixing of tPA [4, 9]	For strongly suspected AIS patients before arrival	Not done
Start tPA on the CT table [4]	Bolus given on CT table	Sometimes
Other procedures after the bolus	For example, thorax X-ray, ECG etc.	Done

The so-called transition period started at the beginning of 2013, and lasted until the end of September 2013. During this period, EPs received education and training in rapid stroke identification, diagnostics including use of the National Institute of Health Stroke Scale (NIHSS), interpretation of head computer tomography (CT) treatment and complications. Nurses and paramedics were trained separately and also together with EPs. The transition period was divided into three parts: Q1/2013 was dedicated mainly to planning the new protocol, Q2 to education and training of the ED staff and Q3 to advanced training in practice. After the transition period, the new protocol became established practice from the beginning of October 2013. The number of patients evaluated by EPs increased during the period since the first 3 months was mainly dedicated to the theoretical education and planning of the protocol.

**Fig. 1:**
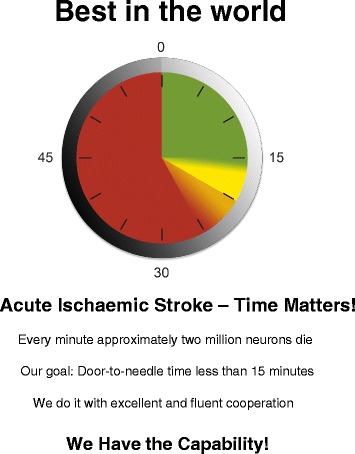
Poster designed to encourage ED staff

Besides education and practical training of nurses and physicians, one important part of the reorganization was motivation of the clinical staff of the ED. This was carried out by way of lectures and group work. Our long-term goal of reaching a median DNT of less than 15 min was also presented. To achieve fast change in our in-hospital process of AIS patients, we used educational background of critical reflexion, which is one of the key components of transformative learning process [11]. The poster is an example of many means we used to get our aim assimilated by all physicians, nurses and radiographers working in our ED (Fig. 1).

### Time delays

Patient data was collected retrospectively from the records. We collected data on all patients who were treated with tPA during 2012 and 2013. The latter year included 9 months of the transition period and the first 3 months of established practice. Door-to-needle time in 2012 was compared with that in 2013. Respectively, OTTs in 2012 and 2013 were compared with each other. Patients with basilar artery occlusion (BAO) or in-hospital stroke were excluded from this analysis.

### Statistical methods

Statistical analyses were performed by using IBM^®^ SPSS^®^ Statistics Version 22 (copyright 2013). Data are presented as median (minimum–maximum) if not mentioned otherwise. Differences in continuous variables were assessed by using the Mann—Whitney *U*-test. A probability value < 0.05 was considered significant.

## Results

From January 2013, EPs acted as the responsible stroke physicians on average 85 % of the time, i.e. 133–147 h per week. A neurologist or the resident neurologist was normally in the ED for three to 5 days a week during office hours.

### Reorganized evaluation and treatment protocol

**Fig. 2:**
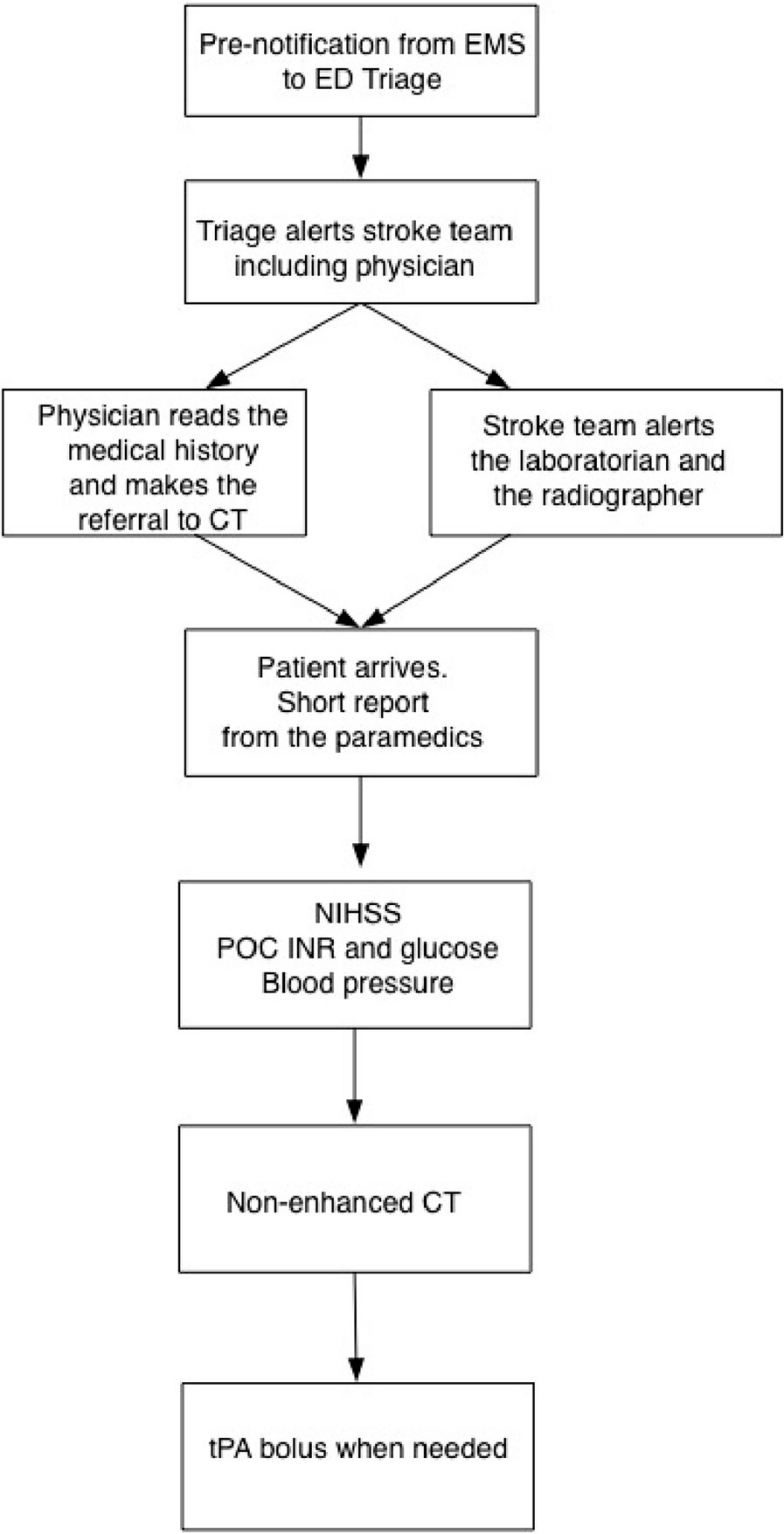
Flow chart of the new in-hospital treatment path for patients with suspected AIS. It is based on scrutinized teamwork led by an EP for most of the time

The new protocol is presented in Fig. 2. The EMS give pre-notification to ED triage when they have a suspected AIS patient under transportation. Suspicion is based on the Face Arm Speech Test [12]. The target time for pre-notification is at least 15 min before arrival so that the team has enough time to be prepared for the upcoming situation. The responsible stroke physician in the ED receives the information by phone or face to face from a nurse immediately after pre-notification. Before that, the triage nurse has already given an automatic simultaneous alarm to the physician and the stroke team. While the patient is still on his/her way to the ED, the physician explores their medical history from electronic patient records in order to exclude contraindications to tPA. At the same time, the stroke team of nurses alerts the laboratory nurse and radiographer. The physician alerts the radiologist on call to be ready to evaluate the head CT scan.

When the patient arrives, the NIHSS score is immediately evaluated. At the same time the laboratory nurse measures the point-of-care (POC) international normalised ratio (INR) and the stroke team nurse measures blood pressure and POC blood glucose. In the case of a privately transported patient, there is no time to prepare beforehand, but otherwise the situation is similar.

After NIHSS scoring the patient is taken immediately to the CT room. A tPA bolus is prepared during imaging or right after CT. In cases of a strong suspicion of AIS without contraindications, the bolus is given while the patient is on the CT table after imaging. A chest X-ray and ECG are carried out after initiation of tPA infusion. If the responsible stroke physician (EP or resident neurologist) needs a second opinion concerning the treatment, he or she consults a neurologist by phone. Hence, the decision is made as soon as possible (Fig. 2). According to our backup system, the stroke physician is always obliged to consult a senior neurologist as well as a radiologist if needed.

### Time delays

The total number of patients treated with tPA was 74 in 2012–2013. Two of them were excluded because of BAO, two because of an in-hospital stroke and six because of missing data. Of the 64 patients included, 31 were treated before and 33 after the start of reorganization. There were 28 men and 36 women. The median age was 72 (33–97) years and median NIHSS score 6 (1–22).

**Fig. 3:**
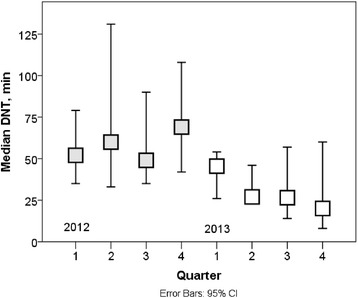
During 2012, i.e. the year before reorganization, the median DNT was 54 (range 34–131) minutes. In 2013, it was 28 (8–61) min. The difference was significant (*p* < 0.001). In the last quarter of 2013, the median DNT was 20 min. In this figure quarterly medians with 95 % confidence intervals are presented

**Fig. 4:**
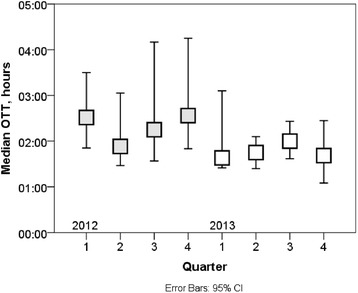
In 2012, the median OTT was 139 (94–265) minutes. In 2013, it was 101 (65–220) min. The difference was significant (*p* < 0.001). In this figure quarterly medians with 95 % confidence intervals are presented

The median DNT decreased from 54 min in 2012 to 28 min in 2013 (*p* < 0.001; Fig. 3). The median OTT also decreased significantly from 2012 to 2013; 139 min vs. 101 min (*p* < 0.001; Fig. 4). During the last 3 months of 2013, the median DNT reached was 20 (8–60) minutes and that of OTT, 101 (65–147) minutes (Figs. 3 and 4).

In 2012, 13 thrombolyses were carried out during office hours and 18 during out-of-office hours. In 2013 the numbers were nine and 24, respectively. The median DNTs during office- and out-of-office hours were 54 and 56 min in 2012, and 30 and 27 min in 2013, respectively.

## Discussion

In this study we present an effectively reorganized evaluation protocol for AIS patients. It is based on well-organized cooperation among clinical staff in the ED and between specialties. Cutting down the DNT as early as during the education and training phase is a meaningful result. Since the specialty is new in our country, this result is very encouraging as regards the development of emergency medicine.

There has been worldwide discussion about the role of EPs in treating acute ischaemic stroke. For example, Kendall encouraged EPs to become involved in the development of thrombolysis protocols [13]. On the other hand, McNamara thought that we are still not ready for community hospital thrombololysis [14].

There have been only a few systematic studies on this specific topic. Smith et al., as early as in 1999, showed that EPs could successfully identify patients with AIS and deliver recombinant tPA with satisfactory outcomes, although the DNT was relatively long [15]. Akins et al. compared neurologists and ED physicians treating stroke with tPA and found that functional outcomes, symptomatic intracerebral bleeding rates and mortality rates were similar in the two groups [16]. Volans noted that stroke thrombolysis can be effectively carried out in a non-specialist district general hospital in the UK [17]. Kaye et al. claimed in 2011 that emergency doctors can be trained rapidly and successfully in provision of thrombolysis for AIS patients, allowing rapid scaling up of the service once the core features are set up [18]. Recently, Lee et al. found no differences between treatments given by a stroke neurologist and another stroke physician [19].

As far as we know, our model is the first in which marked reductions in delays have been achieved by a reform, when EPs have taken the main role in the treatment path. It has to be noted that this was achieved as early as in the 1st year of training and implementation of the new protocol.

According to the results of previous studies, the proportion of AIS patients who have undergone thrombolysis from door to needle in less than 60 min is surprisingly low, on average about 26–29 % of all cases [9, 20]. In recent years, knowledge of the importance of time delays has led to improved outcomes [21, 22]. In our study, this DNT percentage below 60 min was 71 % in 2012. During the reorganization, one patient underwent thrombolysis in 61 min, one in 60 min and the rest (94 %) in less than 60 min in 2013. The hallmark of our new system is that it is available 24/7 all year. While marked improvement of DNTs has occurred, it seems that the quality of patient selection has not suffered. The number of tPA-treated patients was almost the same in 2012 and 2013. There was just one stroke mimic among patients undergoing thrombolysis in 2013 (3.0 %) and the number of cases of symptomatic intracerebral haemorrhage (ICH) or indeed any ICH after iv-tPA was also one (3.0 %) in 2013. In year 2012 the number of any ICHs after tPA was zero (0 %).

We recognize some limitations in our study. Firstly, it is a single-centre study with a relatively small number of patients. Being a middle-size hospital, the number of patients could not be much more during the study period. With reference to Kaye et al., who presented results from the UK in 2011, the number of patients in our study is comparable [18]. Secondly, our study is mainly descriptive, presenting results during the 9-month transition period and the first 3 months of the reorganized system. Outcome results as well as safety data (compared with the Safe Implementation of Thrombolysis in Stroke-Monitoring Study (SITS-MOST), for example) should be main issues in forthcoming studies concerning the impact of our new model [23]. Thirdly, the education period was not formal, which could be a weakness of this study. Although education was given and a neurologist supervised personal training, we did not apply NIHSS certification or other formal procedures, i.e. the training was not formally validated. Besides education in working groups, training was mainly carried out as hands-on training during the first few months of the transition period. The head of the neurology department of our hospital supervised the quality of the education process. Fourthly, the contribution of the EMS was not included in this study. Fifthly, we do not have comprehensive data from the transition period concerning the time points before tPA-bolus, i.e. for example the door-to-imaging time. It seems that this part of the process should be very fluent in order to succeed overall. For this same reason, we cannot make strong conclusions yet regarding the difference between neurologist and EP in the time-points before bolus. It seems that the attitude of changing the process faster and better affects of course to the physicians despite the speciality.

Despite the limitations of this study, our results provide important information for other hospitals developing emergency medicine and dealing with the lack of neurologists on call 24/7. It is possible to successfully reduce DNTs and OTTs by reorganizing the protocol and moving responsibility to EPs.

## Conclusions

In this study we present a new protocol for handling acute ischaemic stroke patients and thrombolysis therapy given by emergency physicians in the emergency department. This came about via scrutinized and good co-operation in our work community. It is possible to achieve shortened door-to-needle time safely by way of this new process led by emergency physicians.
